# Tomographic evaluation of infrazygomatic crest for orthodontic anchorage in different vertical and sagittal skeletal patterns

**DOI:** 10.4317/jced.57267

**Published:** 2020-11-01

**Authors:** Alana Tavares, Iêda-Margarida Crusoé-Rebello, Frederico-Sampaio Neves

**Affiliations:** 1 PhD student in odontology and health - school of dentistry, federal university of Bahia; 2 Associated professor - school of dentistry, federal university of Bahia, division of oral radiology; 3 Adjunt professor - school of dentistry, federal university of Bahia, division of oral radiology

## Abstract

**Background:**

Analysis of the anatomy of the region during preoperative planning is very important in order to minimize the risks of undesired movements in the supporting teeth or even damage to important structures such as the maxillary sinus. To the best of our knowledge, no study evaluated the relationship of these skeletal patterns with the anatomy of the infrazygomatic crest. The aim of this study was to evaluate the tomographic measurements of the infrazygomatic crest for placement of temporary anchorage devices in individuals with different vertical and sagittal skeletal patterns.

**Material and Methods:**

The measurements were analyzed in three regions in the crest of 67 patients above the maxillary first molar: A slice in the long axis of the mesiobuccal root, a slice passing through the center of the furcation area of the tooth, and another slice in the long axis of the distobuccal root. In each of these slices five measurements of the thickness of the infrazygomatic crest were performed, with a difference of 1 mm between them. The sagittal skeletal pattern was determined by the ANB angle and the vertical skeletal pattern by the SN.GoGn angle.

**Results:**

The bone thickness of the crest tended to decrease gradually in the apical direction. There was no difference between different vertical and sagittal skeletal patterns.

**Conclusions:**

The individual parameters did not have significant influence in the thickness of the infrazygomatic crest.

** Key words:**Tomography, X-Ray Computed, orthodontics, mini-implant, infrazygomatic crest, maxilla.

## Introduction

Anchorage control is one of the major challenges in Orthodontics, mainly because of the difficulty in controlling undesired movements in the anchorage units (1). In this respect, the development of skeletal anchorage has been a major advancement, facilitating orthodontic treatment.

Temporary anchorage devices, for example mini-implants and miniplates, are indicated for different therapeutic modalities such as retraction of anterior teeth, extrusion and intrusion of teeth, uprighting molars, indirect anchorage for space closure, and molar mesialization or distalization. In addition, these devices are used for anchorage of skeletal movements in growing patients with a tendency towards Class III malocclusions (2,3). However, instability of these devices can occur depending on the density and thickness of the bone, causing treatment failure (4,5).

The infrazygomatic crest is a pillar of the anterior maxilla. Clinically, the infrazygomatic crest is a palpable bony ridge that runs along the curvature between the zygomatic and alveolar processes. In younger individuals, the infrazygomatic crest is situated between the maxillary second premolar and the first molar, while in adults this structure is found above the maxillary first molar (6).

A possible site in the maxilla where mini-implants or miniplates can be placed for skeletal anchorage is the region of the infrazygomatic crest because it consists of one bone, with two cortical layers (buccal and floor of the maxillary sinus). The anatomical advantage of this site is bicortical fixation, which could increase primary stability of the miniscrew (6). Computed tomography (CT) has been used to evaluate the thickness of the infrazygomatic crest and to determine the best site for the insertion of mini-implants that would avoid perforation of the maxillary sinus and damage to the dental roots (6-8).

The correct analysis of the anatomy of the region during preoperative planning is very important in order to minimize the risks of undesired movements in the supporting teeth or even damage to important structures such as the maxillary sinus. To the best of our knowledge, no study evaluated the relationship of these skeletal patterns with the anatomy of the infrazygomatic crest. Therefore, the objective of the present study was to evaluate the thickness of the infrazygomatic crest using CT scan for placement of temporary anchorage devices in subjects with different vertical and sagittal skeletal patterns.

## Material and Methods

The project was approved by the Ethics Committee of the School of Dentistry, Federal University of Bahia (Protocol 43745915.9.0000.5024).

Multislice CT images from 67 patients (134 sides) obtained from the image database of the School of Dentistry, were evaluated, including 40 (59.7%) from female patients and 27 (40.3%) from male patients. All participants provided a written informed consent.

All multislice CT images, in the database, with field of view (FOV) of full face of patients older than 18 years were included. Tomographies images showing suggestive signs of facial trauma or fracture and patients with a history of facial surgery, with signs of maxillary tumors, with syndromes, with cleft lip/palate, and with severe asymmetries were excluded. Patients with absence of any maxillary teeth and those with miniscrews in the region of the infrazygomatic crest were also excluded.

The images were acquired with a 64-channel multislice tomograph (Light Speed VCT, GE Healthcare Bio-Sciences,Piscataway, NJ, USA), operating at 120 kV and 200 mA. The axial slices were obtained at a thickness/increment of 0.6 mm each, with an FOV of 32 cm (full face), and evaluated with an iMac computer (27 inches, 2560 x 1440; Apple, Inc., Cupertino, CA, USA) using the Osirix v.3.9.3 software (Pixemeo, Geneva, Switzerland). The measurements were made by a properly calibrated examiner with experience in CT, who could apply all tools necessary for better evaluation of the images, such as those used to alter brightness and contrast and zoom.

The measurements were obtained using an adaptation of the method proposed by Baumgaertel and Hans (7), in which the following three tomographic slices, on both sides, in the axial, coronal and sagittal orientation, perpendicular to the buccal bone and parallel to the long axis of the maxillary first molar were selected: one slice in the long axis of the mesiobuccal root of the maxillary first molar, one in the long axis of the distobuccal root of the maxillary first molar, and another slice passing through the center of the furcation area of the maxillary first molar.

**Figure 1:**
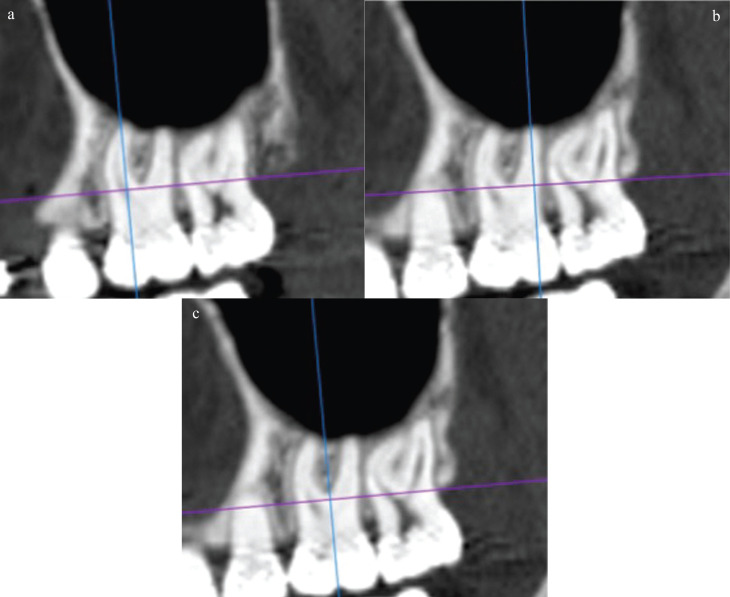
a. Sagittal slice showing the long axis of the mesiobuccal root. b. Sagittal slice showing the long axis of the distobuccal root. c. Sagittal slice showing the long axis of the tooth.

**Figure 2:**
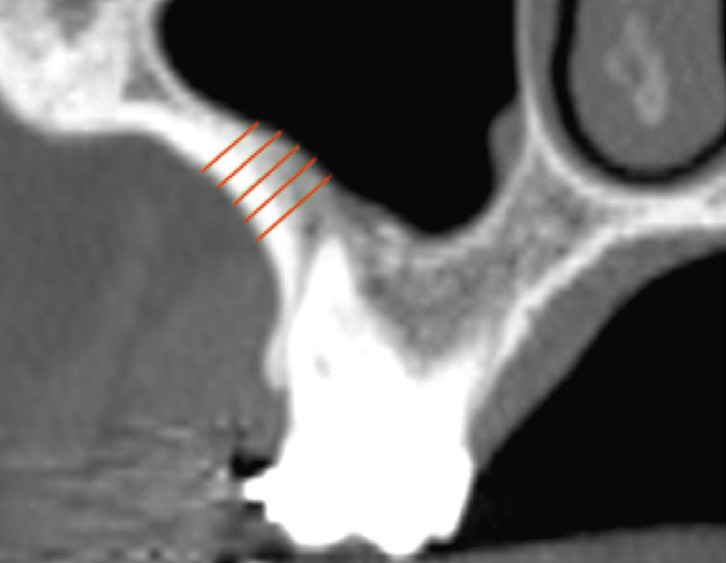
Measurement of the bone thickness of the infrazygomatic crest.

Five measurements of the thickness of the infrazygomatic crest were obtained in each of these slices in the coronal plane. The first measurement along the long axis of the mesio or distobuccal roots was made perpendicular to the buccal bone of the infrazygomatic crest, 2 mm from the root apex. In the center of furcation area, the first measurement was made 2 mm above the palatine and buccal roots, choosing the highest root. In the case of invagination of the maxillary sinus in the region, the measurement was only obtained from the buccal roots. The four subsequent measurements were obtained adding 1 mm in the cranial direction (Figs. 1,2). For analysis of intraexaminer reproducibility, 20% of the sample was reevaluated after 30 days.

In the CT images the sagittal skeletal pattern was determined according to the classification of Steiner (9) based on the ANB angle (angle formed by the A point, nasion and B point), which defines the sagittal relationship of the jaws. An ANB angle of 0° to 4° is classified as Class I skeletal pattern, > 4° as Class II, and < 0° as Class III.16 Twenty (29.9%) of the 67 patients were classified as Class I, 32 (47.7%) as Class II and 15 (22.4%) as Class III. The vertical skeletal pattern was established according to the classification of Riedel (10). The facial types were divided based on the SN.GoGn angle into mesofacial (27° to 37°), brachyfacial (< 27°), and dolichofacial (> 37°). Twenty-three (34.3%) of the patients were classified as dolichofacial, 30 (44.8%) as mesofacial, and 14 (20.9%) as brachyfacial.

The data were analyzed with the Minitab® 14.20 software (State College, PA, USA). The intraclass correlation coefficient (ICC) was applied to evaluate intraexaminer reproducibility, which was classified as excellent (1.0 to 0.81), substantial (0.80 to 0.61), moderate (0.60 to 0.41), reasonable (0.40 to 0.21), or poor (0.20 to 0.0).

The measurements made in the infrazygomatic crest were compared according to side, gender and sagittal and vertical skeletal pattern by analysis of variance (ANOVA) with Tukey’s post hoc test, adopting a level of significance of 5%.

## Results

The ICC values showed excellent intraexaminer reproducibility (0.90) for all reevaluated measures.

Table 1 summarizes the data according to side and gender. The mean values of bone thickness were similar between sides and genders, with no significant difference in any of the measurements (*p*>0.05). However, the bone thickness of the crest tended to decrease gradually in the apical direction at all sites evaluated.

**Table 1:**
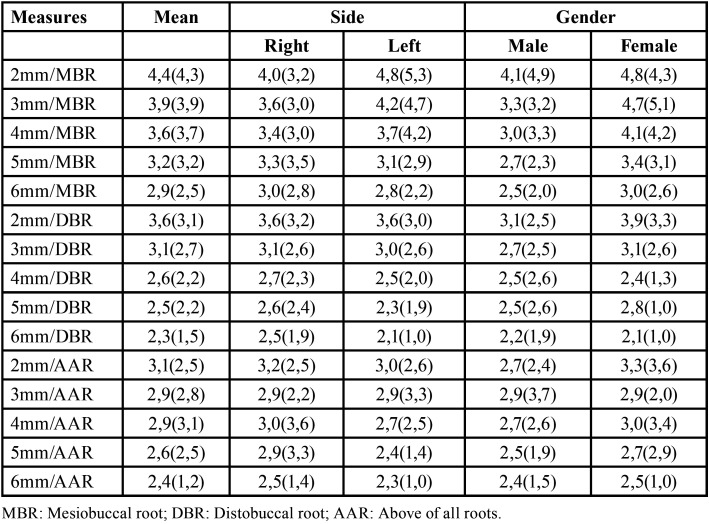
Mean (standard deviation) thickness (mm) of the infrazygomatic crest according to gender and side.

With respect to sagittal skeletal pattern (Table 2), no significant difference between skeletal classes was observed, regardless of gender (*p*>0.05). The lowest and the highest thickness of the infrazygomatic crest was observed in Class I males at 5 and 6 mm above distobuccal root (1.6mm) and in Class I and III females at 3 mm above the mesiobuccal root (5.9 mm), respectively.

**Table 2:**
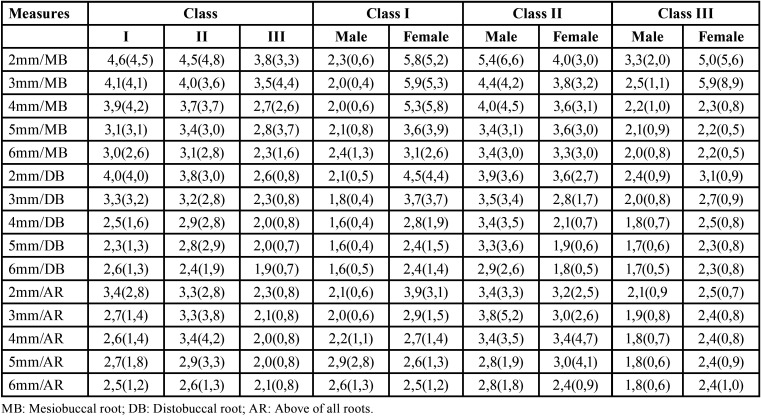
Mean (standard deviation) ithickness (mm) of the infrazygomatic crest according to gender and sagittal skeletal pattern.

According to the vertical skeletal pattern (Table 3), it was observed no difference between the brachyfacial, mesofacial and dolichofacial groups, regardless of gender (*p*>0.05). The lowest and the highest thickness of the infrazygomatic crest was observed in dolichofacial males at 5 and 6 mm above the mesiobuccal root (1.8 mm for both) and dolichofacial famales at 2 and 3 mm above the mesiobuccal (5.9 mm), respectively.

**Table 3:**
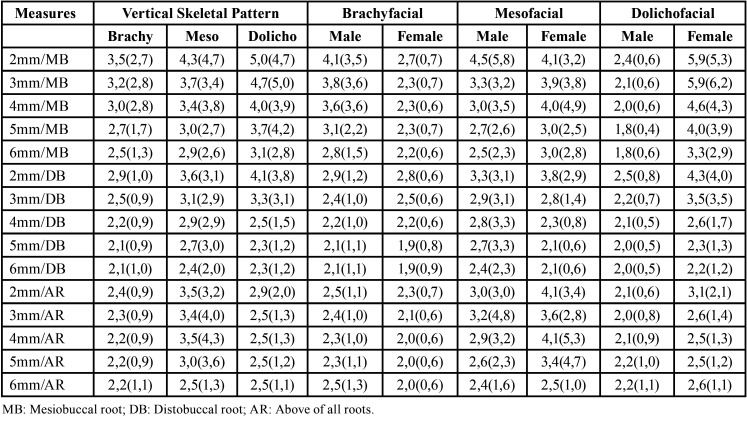
Mean (standard deviation) thickness (mm) of the infrazygomatic crest according to gender and vertical skeletal pattern.

## Discussion

Previous studies using CT have shown that the vertical and sagittal skeletal patterns are related to different bone structures such as the pterygomaxillary region (11), alveolar and cortical bone (12-14). The present study showed that the bone thickness of the infrazygomatic crest is related to individual parameters that should be evaluated carefully before orthodontic treatment planning.

Since most manufacturers offer mini-implants of different lengths, the smallest of them 6 or 7 mm long (7), and screws for miniplate fixation measuring 4 to 7 mm in length (8), perforation of the maxillary sinus or nasal cavity is possible depending on the thickness of the infrazygomatic crest (7). The consequence in some cases is sinusitis or mucocele (15). A thicker bone permits the use of longer screws, greater bone contact, and better primary stability (16).

In the present study, the bone thickness of the crest tended to decrease gradually in the apical direction in accordance with other studies (7). The greatest mean bone thickness of the crest was observed in the region 2 mm above the mesiobuccal root, with 4.4 mm. These results show sufficient bone thickness at this site for the placement of mini-implants, minimizing the risk of perforation of the maxillary sinus. Liu *et al.* (18), evaluating bone thickness between the buccal roots of the maxillary first molar, found the greatest value of 3.05 mm at a distance of 11 mm from the alveolar crest. Thus, in contrast to the present study, the more distant from the alveolar crest, the greater the bone thickness. Liou *et al.* (6) and Chapada *et al.* (19) suggested that, to obtain a bone thickness of 6 mm in the infrazygomatic crest, mini-implants should be inserted above the maxillary first molar, 14 to 16 mm above the maxillary occlusal plane, at an angle of 55° to 70° from this plane. This method can be used when there is smaller bone thickness.

As observed in other studies (6,17), we found no significant differences between the right and left sides, demonstrating the absence of severe facial asymmetry in the patients studied. In addition, gender did not influence the anatomical measures of the infrazygomatic crest, in agreement with previous studies (17,20).

According to Sadek *et al.* (13,14), dolichofacial individuals exhibit lower alveolar and interradicular cortical bone thickness in the anterior region. Consequently, the risk of movement of the incisors in the anteroposterior direction is higher. Bajracharya (12) also found lower alveolar bone thickness in the region of the maxillary incisor in dolichofacial patients. However, the site evaluated in this study was the infrazygomatic crest and mean bone thickness was similar between the different vertical facial patterns (*p*>0.05). The greatest mean thickness was found in dolichofacial patients at the most coronal site from the mesiobuccal root area (5 mm). Similarly, Chen *et al.* (21) also observed no difference between vertical pattern groups when they evaluated the infrazygomatic crest 7 mm above the alveolar crest of the mesiobuccal root of the maxillary first molar of Class II patients.

Despite the literature states, there is an association between cranial bone thickness and different sagittal skeletal patterns (22), the present results showed no significant difference between the groups. The greatest mean thickness was found in Classe I patients at the most coronal site from the mesiobuccal root area (4.6 mm). In a study using lateral x-ray, Endo *et al.* (23), also, did not found significant difference between the size of the maxillary sinus and the sagittal relationship of the jaws.

Lee *et al.* (8) evaluated the bone thickness of the infrazygomatic crest in Class III growing patients. In these patients, the superior and lateral areas of the zygomatic process of the maxilla were thicker (thickest point on average 5 mm and thinnest point 1.1 mm), in contrast to the results of the present study. This difference might be explained by some methodological differences between the two studies. In the study of Lee *et al.* (8), patients in the phase of bone growth (10 to 13 years) were studied and measurement of the infrazygomatic crest was started at the inferior border of the zygomatic process of the maxilla. In the present study, the measurements were made in a more inferior area (above the buccal roots and furcation) in adults.

The risk of cortical bone perforation in the maxillary sinus and the primary stability of the mini-implants shows the need of an accurate preoperative planning for the placement of temporary anchorage devices. In this respect, bone thickness and density are important factors for the success of this intervention (4,5). According to Kim & Kim (24) the insertion of mini-implants less than 1mm from the surface of the dental root causes its resorption. Jia, Chen and Huang (25) found slight membrane thickening and bone resorption when the sinus is perforated. The authors recommend insert the mini-implant through double cortical bone plates for a better primary stability but limiting the insertion depth to 1 mm. These facts highlight the need to have an adequate minimum bone thickness for the placement of mini-implants. The results in our study showed the need for greater preoperative analysis, being able to use the inclination of the screws.

According with our results, the individual parameters (side, gender, vertical and sagittal skeletal patterns) did not have significant influence in the thickness of the Infrazygomatic crest. The anatomical knowledge associated with a surgical and imaging planning are necessary in order to avoid complications such as perforation of the maxillary sinus during insertion of temporary anchorage devices.
